# Genome-Wide Association Study of Body Weights in Hu Sheep and Population Verification of Related Single-Nucleotide Polymorphisms

**DOI:** 10.3389/fgene.2020.00588

**Published:** 2020-07-03

**Authors:** Yuhao Cao, Xuemei Song, Huili Shan, Junfang Jiang, Pei Xiong, Jianliang Wu, Fangxiong Shi, Yongqing Jiang

**Affiliations:** ^1^College of Animal Science and Technology, Nanjing Agricultural University, Nanjing, China; ^2^Institute of Animal Husbandry and Veterinary, Zhejiang Academy of Agricultural Sciences, Hangzhou, China; ^3^Department of Biochemistry and Molecular Biology, Zhejiang Key Laboratory of Pathophysiology, Medical School of Ningbo University, Ningbo, China

**Keywords:** Hu sheep, body weight, GWAS, population verification, transcriptional activity

## Abstract

Body weight (BW) is a critical economic trait for meat production in sheep. The current study aimed to perform a genome-wide association study (GWAS) to detect significant single-nucleotide polymorphisms (SNPs) that are associated with BW in Hu sheep. The comparison and analysis of the G1 and G2 generations of a nucleus meat Hu sheep breeding herd revealed four SNPs identified by GWAS. The subsequent verification of the significant SNP loci in the Hu sheep G3 generation nucleus herd also detected nine SNPs in significant SNP regions. Two SNPs were significantly associated with the BW of Hu sheep (*p* < 0.05). OARX_76354330.1 and s64890.1 could be identified as functional SNPs for the growth traits of Hu sheep. *CAPN6*, as a candidate gene, was significantly different in the biceps femoris and longissimus dorsi muscles of weaning (60-day) and 6-month sheep, which facilitated the discovery of causal variants for BW and contributed to the marker-assisted selection breeding of Hu sheep.

## Introduction

In sheep, body weight (BW) is a critical economic trait for meat production (Gebreselassie et al., 2019). Genome-wide association studies (GWAS) have been applied to identify candidate genes for many quantitative traits. This provided an opportunity to increase the selection efficacy, especially for the traits that cannot be easily improved by conventional selection methods (Dekkers, 2004). After multiple quantitative trait loci (QTL) were used for production traits in cattle, some QTL studies have been conducted in sheep (Margawati et al., 2006). Development of high-throughput single-nucleotide polymorphism (SNP) genotyping technologies led to the use of GWAS to detect candidate genes for the quantitative traits, which further enhanced the efficiency of animal breeding programs. Although important candidate genes have been identified by GWAS in different species, only a few QTL studies have been conducted in sheep. To date, there are 753 sheep data points in the animal QTL database (QTLdb^1^) (Hu et al., 2013). The GWAS study of the Scottish Blackface lamb identified QTL on OAR1, 3, 24, and especially on OAR6, that are associated with the effects on muscle, fat, and bone traits (Matika et al., 2016). A previous study found that 39 SNPs could affect the birth weight of the Australian Merino sheep. Among these, 13 SNPs and OAR6_41936490.1 were significantly associated with birth weight (Al-Mamun et al., 2015). In addition, OAR384073899.1 on intron 8 of the *CAMKMT* gene can significantly affect the daily weight gain of Ujummu sheep after weaning (Ma et al., 2016), and 10 SNPs including OAR8_75441328.1, OAR17_34475530.1, s58995.1, and 329 were significantly associated with daily weight gain after weaning of the resource group consisting of Sunit sheep, German Mutton Merino, and Dorper sheep (Zhang et al., 2013). In the correlation analysis of wool weight, three chromosome-wide significant associations were detected for SNPs on chromosomes 17 and 20 affecting greasy fleece weight across four shearings in a Baluchi sheep population (Ebrahimi et al., 2017). *FOS like 2* or *AP-1* transcription factor subunit (*FOSL2*) was significantly associated with BW in Luzhong mutton sheep (Tao et al., 2020). Moreover, *RAB6B*, *Tf*, and *GIGYF2*, could be considered as candidate genes for the analysis of the birth weight of Lori-Bakhtiari sheep (Ghasemi et al., 2019). Birth weight is the earliest available BW trait with considerable impact on the ability of lamb survival and growth performance traits (Ptáček et al., 2017). BW measured at birth or other life stages in the meat-producing livestock is a major indicator of productivity, growth, health, and preventive management.

There is no GWAS report on Hu sheep. Hence, the present study aimed to (1) perform a GWAS to detect significant SNPs that are associated with the birth weight, weaning weight, 6-month weight, and yearling weight, using the data from 240 Hu sheep genotyped with the Illumina Ovine SNP50 BeadChip; (2) verify significant SNPs in the progeny of Hu sheep; (3) study the expression differences of candidate genes during sheep growth.

## Materials and Methods

### Study Population

Since 2006, the Herbivore Livestock Research Group of the Institute of Animal Husbandry and Veterinary Medicine, Zhejiang Academy of Agricultural Sciences, has established two Hu sheep nucleus herds in Huzhou and Hangzhou. The 240 Hu sheep selected for the study were from the parent (G1) and progeny (G2) of the Hu sheep nucleus herd Culture Department in Huzhou Taihu Lake Culture Cooperative. A total of 202 females of the G3 generation nucleus herd in Hangzhou were used to verify significant SNPs. The feeding management of two Hu sheep nucleus herds was based on the same method applied to meat sheep.

### Phenotypic Data and DNA Extraction

BW measurements, including birth weight, weaning weight, 6-month weight, and yearling weight, were taken in a quiet, relaxed state in the two nucleus herds of Hu sheep. To reduce manmade errors in BW measurements, each sheep was measured at least twice by the same person, and the average was the final measurement. Blood samples were collected from the posterior venous plexus of Hu sheep to extract genomic DNA using the standard phenol/chloroform method (Russell and Sambrook, 2001).

### Genotyping and Quality Control

Genotyping of individual SNPs was performed using the Ovine SNP50BeadChip (Illumina Inc., San Diego, CA, United States). Subsequently, the data were entered into Bead Studio software for related analysis. Chips with call rate <0.99 were removed from further analysis. After the genotypes of the software output were sorted and proofread, statistical analysis was performed, and the inaccurate SNP loci were eliminated or corrected by the software to obtain the characteristics of the genome-wide data. The nucleic acids were typed using a genome-wide genotyping system, and the data were visualized into genotyping results using the Genome Studio software, saved in.txt format, and exported.

### Population Structure Analysis and Genome-Wide Association Study

Population structure analysis was performed using admixture v1.3. For kinship plot, a heat map of the values in the kinship matrix was created. Kinship matrix was analyzed using the TASSEL5.0. PLINK 1.90p was used to pre-filter the SNP data. Biallelic sites with the maximum genotype-missing rate of 0.2, and minimum minor allele frequency (MAF) of 0.05 were retained. Missing genotypes were imput using beagle 5.1 (version: 24Mar20.5f5). The imputed data were then screened using the LD-based variant pruner and haplotype block estimator implemented in PLINK 1.90p with a windows size of 50 kb, step size of 10, and a pairwise *r*^2^ threshold of 0.2. Thereafter, genotypic data were processed for quality control. The mixed linear model (MLM) of TASSEL 5.0 software^2^ was used for GWAS analysis of SNPs, and the SNPs related to the phenotype of the Hu sheep core traits were mined. The MLM model calibrates three confounding factors: gender, group structure, and kinship. The specific model is

Y=X⁢β+S⁢α+Q⁢ν+Z⁢u+e,

where *Y*: phenotypic value vector of the weight trait of the core group of Hu sheep; β: a fixed effect vector other than the SNP and the population structure; α: SNP effect vector; *v*: group structure effect vector; *u*: multi-gene background effect vector; *e*: residual effect vector; *X*, *S*, *Q*, and *Z*: correlation matrices of β, α, *v*, and *u*, respectively.

While performing the correlation analysis of the traits of the nucleus herd of Hu sheep, if there are errors in the multiple hypothesis tests, it is necessary to analyze and correct the *p* value. The *F* and *p* values were calculated by MLM and then tested. The specific formula is as follows:

$$P_{S}=\frac{\alpha }{N}$$

α: level of significance; *N*: number of independent SNPs used in the analysis. After genome-wide association analysis to obtain significant SNPs, the 500 bp sequence upstream and downstream of the SNP locus was downloaded and aligned against the sequences from databases, such as NCBI and *Ovis aries*_v4.0 (UCSC)^3^ to determine SNP locations and the adjacent genetic information for designing primers for PCR and direct sequencing (Supplementary Table S1).

### Population Verification of Candidate Functional SNPs for BW Traits in Hu Sheep

A total of four SNPs were associated with the weight trait of G1 and G2 generations of the nucleus herd of Hu sheep. The sequences around the four SNPs were amplified to detect additional SNPs for further population genetics and association analyses between the SNPs and the BW traits of 202 female Hu sheep G3 generation nucleus herd.

The PCR reaction was performed in a volume of 20 μL containing 1 μL of genomic DNA, 10 μL of 2 × Tiosbio^®^ Aurora Master Mix (Tiosbio, Beijing, China), 0.2 μL of 10 μM forward primer, and 0.2 μL of 10 μM reverse primer. The amplification reaction was pre-denaturation at 94°C for 3 min; 35 cycles of denaturation at 94°C for 25 s, annealing at 55°C for 25 s, extension at 72°C for 20 s; extension at 72°C for 10 min, followed by storage at 4°C. Mutation Surveyor 5.02 (SoftGenetics, LLC, State College, PA, United States) was used to discover the SNPs of each individual by Sanger sequencing. PopGen32 software was employed to calculate the gene frequency, genotype frequency, polymorphism information content (PIC), Shannon information content (SIC), and site heterozygosity (H) of each SNP. The GLM procedure in SPSS 19.0 software (SPSS, Inc.) was performed to evaluate the SNP-phenotype association of G3 generation nucleus herd.

### Detection of Differences in Expression of Candidate Genes in Skeletal Muscle During the Growth of Hu Sheep

Candidate functional genes associated with BW of sheep were selected. RNA from biceps femoris muscle and longissimus dorsi muscle in weaning (60-day) and 6-month sheep was extracted using an animal tissue total RNA kit (Simgen, China), and reverse transcription was performed using a Polestar first cDNA synthesis kit + gDNA removal (Tiosbio, Beijing, China). Quantitative PCR was conducted using the KOD SYBR qPCR Mix (Toyobo, Japan) for the detection and quantification of the expression differences of candidate genes in the skeletal muscle of weaning and 6-month sheep.

The corresponding primer pairs were listed in Supplementary Table S2.

## Results

### Descriptive Statistics and Quality Control

The genomic DNA was detected by the whole genome-based detection platform, and the quality control management was performed on 240 individual samples and 54241 SNP sites using Plink1.90 software. We excluded the following: (1) 3577 SNPs with call rate <90%; (2) 20 SNPs not in accordance with the Hardy–Weinberg equilibrium; (3) 4435 SNPs with a minor allele frequency <0.05. Also, 14 individuals were excluded because of the call rate <99%. After quality control, 226 sheep and 35,363 independent SNPs were used for the final analysis.

### Population Structures and Association Analyses

**FIGURE 1:**
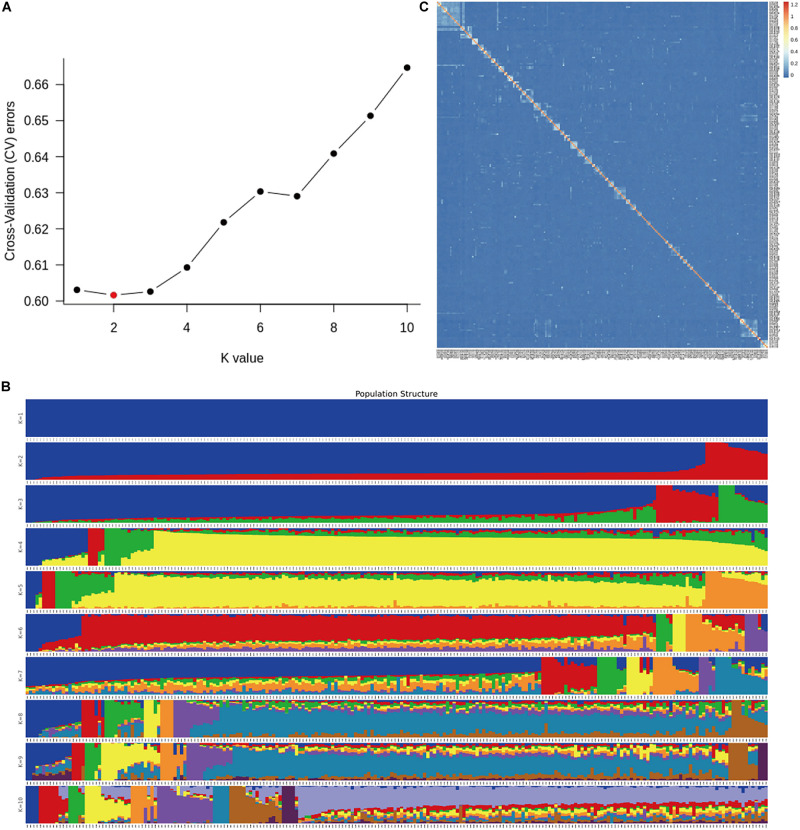
Population structure analysis in the G1 and G2 generations nucleus herd of Hu sheep. **(A)** Population structure of 226 Hu sheep with optimal K = 2; **(B)** Population structure of 226 Hu sheep with K from 1 to 10; **(C)** Kinship plot of 226 Hu sheep.

**FIGURE 2:**
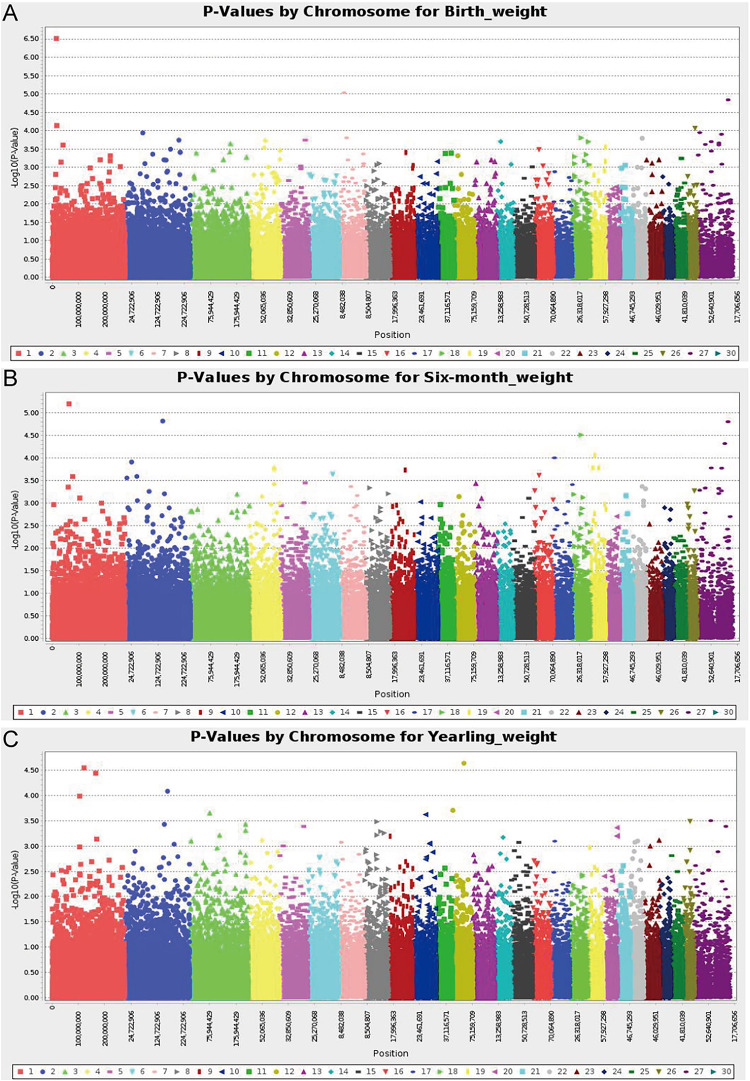
Manhattan plot of analysis results; -log_10_ (*p* values) in the studied population of Hu sheep. **(A)** Manhattan plot of the results of the birth weight analysis; **(B)** Manhattan plot of the results of the 6-month weight analysis; **(C)** Manhattan plot of the results of the yearling weight analysis.

A total of 226 Hu sheep were randomly selected from the group of the Huzhou Taihu Lake Culture Cooperative. According to the population structure, the result was given by admixture v1.3 with K from 1 to 10, the optimal K was 2 (Figure 1A). Kinship estimation of all individuals indicated the effectiveness of sampling (Figures 1B,C). GWAS identified four SNPs significantly affecting the birth weight, 6-month weight, and yearling weight of Hu sheep. Based on the number of independent effective SNPs, 1.41 × 10^–6^ and 2.28 × 10^–5^ were regarded as genome-wide and suggestive significant values, respectively. The results show that four SNPs were significantly correlated with birth weight. s37755.1 (*p* = 3.01 × 10^–7^) was located on chromosome 1, s39389.1 (*p* = 9.31 × 10^–6^) on chromosome 7, and OARX_76354330.1 (*p* = 1.45 × 10^–5^) on chromosome 27. One SNP was significantly correlated with the 6-month weight. The most significant SNP was OARX_76354330.1 (*p* = 1.56 × 10^–5^) located on chromosome 27. In addition, one SNP was correlated with the yearling weight: s64890.1 (*p* = 2.31 × 10^–5^) located on chromosome 12; it was close to the threshold value, so it was also included in genome-wide suggestive SNPs, and done with subsequent population verification (Figure 2). There was no SNP significantly correlated with the weaning weight of Hu sheep.

Three SNPs obtained from GWAS analysis occurred within a gene or in a regulatory region near a gene: s37755.1 in *SCMH1*, OARX_76354330.1 in *CAPN6*, and s39389.1 in *ITGA11*. In addition, one SNP locus was significantly associated with genome-wide levels in the intergenic region: s64890.1 located 364k bp downstream of *CAMTA1* (Table 1).

**TABLE 1:** Analysis of related SNPs associated with birth weight of Hu sheep.

Trait	Related SNPs	Chr	Position (bp)	*p*-value	Nearest gene distance^#^ (bp)
Birth weight	s37755.1	1	15359859	3.10E-07	*SCMH1* within
	s39389.1	7	14990248	9.31E-06	*ITGA11*
	OARX_76354330.1	27	117253931	1.45E-05	*CAPN6* within
Six-month weight	OARX_76354330.1	27	117253931	1.56E-05	*CAPN6* within
Yearling weight	s64890.1	12	156496206	2.31E-05	*CAMTA1* -364270

**p*-values calculated from the mixed linear model analysis. ^#^Positive value denotes the gene location downstream of SNP; negative value denotes the gene location upstream of SNP.*

### Population Verification of Candidate Functional SNPs

**FIGURE 3:**
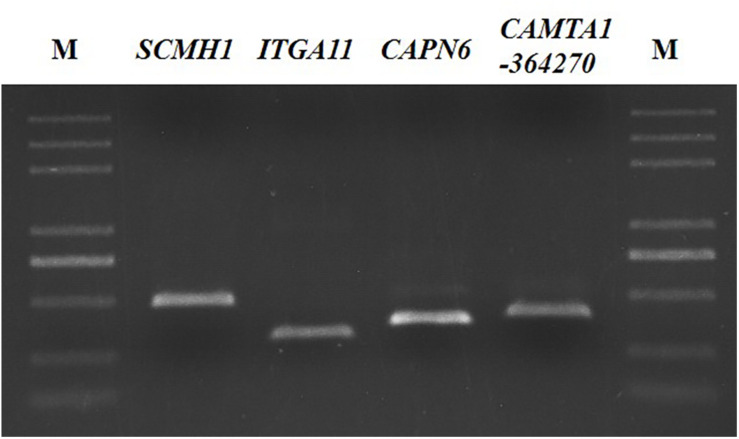
PCR amplification products of BW trait-related SNPs in sheep. M: DL2000plus.

GWAS showed that the four SNPs were associated with BW in the Hu sheep G1 and G2 generation nucleus herd. Subsequently, while verifying the significant SNP loci in 202 females from the G3 generation nucleus herd, an additional 9 SNPs were detected in proximity to the significant SNP loci amplification products (Figure 3). All loci were subjected to genotyping, population genetic analysis, and the association analysis between SNPs and BW.

A total of 13 SNPs were detected. All GWAS-predicted SNPs were individually detected, and some other mutations were identified near the target SNP loci in the amplification products (Supplementary Table S3). The amplification product of OARX_76354330.1 detected two mutations, and the amplification product of s39389.1 contained three mutations. Furthermore, the amplification products of s64890.1 and s37755.1 detected four mutations, respectively. Moreover, five SNPs presented low polymorphisms (PIC < 0.25), and the other eight SNPs had moderate polymorphisms (0.25 < PIC < 0.5) (Supplementary Tables S4, S5).

Two SNPs were significantly associated with birth weight of Hu sheep females: T > A mutation at OARX_76354330.1 and A > G mutation at 28 bp downstream of OARX_76354330.1 (Table 2). One SNP was significantly associated with weaning weight of Hu sheep females: T > C mutation at s39389.1 (Table 3). One SNP was significantly associated with yearling weight of Hu sheep females: G > A mutation at s64890.1 (Table 4).

**TABLE 2:** Association analysis between SNPs and birth weight of Hu sheep.

SNP loci	Genotype	Numbers	Birth weight (kg)
T > A mutation at OARX_76354330.1	TT	121	2.9 ± 0.3^b^
	TA	76	2.8 ± 0.3^b^
	AA	6	3.2 ± 0.3^a^
A > G mutation at 27 bp downstream of OARX_76354330.1	AA	121	2.9 ± 0.3^b^
	AG	77	2.8 ± 0.3^b^
	GG	5	3.3 ± 0.3^a^

*Values with the same superscript for the same column have no significant difference (*p* > 0.05). Values with different superscript for the same column have significant differences (*p* < 0.05).*

**TABLE 3:** Association analysis between SNPs and weaning weight of Hu sheep.

SNP loci	Genotype	Numbers	Birth weight (kg)
T > C mutation at s39389.1	TT	172	17.8 ± 2.4^a^
	TC	23	16.7 ± 1.6^b^

*Values with the same superscript for the same column have no significant difference (*p* > 0.05). Values with different superscript for the same column have significant differences (*p* < 0.05).*

**TABLE 4:** Association analysis between SNPs and yearling weight of Hu sheep.

SNP loci	Genotype	Numbers	Yearling weight (kg)
G > A mutation at s64890.1	GG	117	62.6 ± 9.8^a^
	GA	69	60.5 ± 8.8^b^
	AA	17	58.1 ± 9.2^b^

*Values with the same superscript for the same column have no significant differences (*p* > 0.05). Values with different superscript for the same column have significant differences (*p* < 0.05).*

### Differential Expression of Candidate Genes in Skeletal Muscle During the Growth of Hu Sheep

The expression of the *CAPN6* gene was significantly different in the biceps femoris and longissimus dorsi muscles of weaning (60-day) and 6-month sheep (*p* < 0.05), but the difference in the expression of *ITGA11* or *SCMH1* was not significant (Table 5).

**TABLE 5:** Detection of candidate genes at different ages.

Candidate genes	Age	Biceps femoris muscle (2^–△^^△^^ct^ value)	Longissimus dorsi muscle (2^–△^^△^^ct^ value)
*CAPN6*	Weaning (60-day) sheep	(2.5 ± 0.2) × E-6a	(1.5 ± 0.2) × E-6^a^
	Six-month sheep	(1.6 ± 0.5) × E-6b	(0.8 ± 0.1) × E-6^b^
*ITGA11*	Weaning (60-day) sheep	(1.6 ± 0.1) × E-6a	(1.2 ± 0.1) × E-6^a^
	Six-month sheep	(1.8 ± 0.6) × E-6a	(1.3 ± 0.4) × E-6^a^
*SCMH1*	Weaning (60-day) sheep	(4.2 ± 2.0) × E-8a	(3.0 ± 0.7) × E-8^a^
	Six-month sheep	(1.6 ± 0.5) × E-8a	(5.7 ± 4.7) × E-8^a^

*Values with the same superscript for the same column have no significant differences (*p* > 0.05). Values with different superscript for the same column have significant differences (*p* < 0.05).*

## Discussion

BW is a quantitative trait with moderate-to-high heritability (Buzanskas et al., 2014). In this study, we performed a GWAS on Hu sheep by genotyping data using an OvineSNP50 Genotyping Bead chip that included about 50,000 SNPs. The analysis identified four SNPs that were associated with BW. Population stratification can cause false positives in GWAS analysis. Although this phenomenon is significantly correlated with BW, it is only falsely associated with the related traits (Lander and Kruglyak, 1995). In the sampling group, because the test sheep were from the same semi-open nucleus herd, group stratification was not applicable. The rationality of using groups in this study was illustrated by population structure analysis and kinship matrix diagram. Notably, the GWAS results showed that OARX_76354330.1 was not only related to birth weight but also 6-month weight. This result strongly suggests that OARX_76354330.1 can be used as a functional candidate SNP for Hu sheep BW traits.

s37755.1 is located within the SCMH1 (GeneID: 101103519). Scmh1 is a mammalian homolog of the Drosophila Sex comb on midleg (Scm) gene (Tomotsune et al., 1999). Scmh1 mediates the molecular interaction between Polycomb-group complex 1 and geminin (Luo et al., 2004) and plays an important role in maintaining the activity of hematopoietic stem cells (HSCs) (Yasunaga et al., 2013). SCMH1 is involved in cell fate and cell cycle and also plays an important role in regulating bone development. Skeletal abnormalities and male infertility were observed in mutant mice lacking the Scmh1 SPM domain (Takada et al., 2007). *SCMH1* has been shown to upregulate the expression of several bone biomarkers, including alkaline phosphatase (ALP), collagen type 1 (COL-1), osteocalcin (OCN), osteopontin (OPN), and dwarf-related transcription factors 2 (RUNX2), in the mouse osteoblast MC3T3-E1 cells, highlighting its regulatory role in bone formation (Pei et al., 2019).

s39389.1 is located within the *ITGA11* (GeneID: 101105162). *ITGA11* belongs to the integrin family. Integrin α11 was overexpressed in the stroma of most head and neck squamous cell carcinomas (HNSCC) and correlated positively with alpha smooth muscle actin expression (Parajuli et al., 2017). ITGA11 is a research hotspot in cancer research, such as lung and breast cancers. ITGA11 plays a significant role in cancer migration and invasion, leading to higher recurrence (Smeland et al., 2019; Wu et al., 2019).

OARX_76354330.1 is located within the *CAPN6* (Gene ID: 101110122). Calpain 6 (*CAPN6*) is a calcium-dependent intracellular lysosomal protease. CAPN6 is a non-proteolytic protease with microtubule binding and stabilizing activities that promotes cytoskeletal structure and microtubule stability in osteoclasts (Hong et al., 2011). *CAPN6* expressed in embryonic tissue can be used as a microtubule stabilizing protein and is involved in the regulation of microtubule dynamics and cytoskeletal organization (Tonami et al., 2007, 2011). *CAPN6* is also involved in the proliferation and apoptosis of uterine leiomyoma cells and plays a regulatory role in the development of the nervous system of rats (Su et al., 2019; Zhu et al., 2020).

The four SNPs detected by GWAS were verified in the Hu sheep G3 generation nucleus herd, and an additional nine SNPs were detected. The OvineSNP50 BeadChip is the most comprehensive genome-wide genotyping array for the ovine genome; however, the SNP density of the GWAS chip is insufficient, and many SNP sites cannot be detected. Therefore, subsequent population verification is indispensable. The population verification is not only an effective supplement to the GWAS analysis results but also finds new SNP sites during the verification. GWAS can be used as an important tool for screening candidate functional genes and SNPs. It can provide a road sign–like effect and indicate the research direction for subsequent verification without overwhelming the researchers with a vast array of sequence information.

T > A mutation at OARX_76354330.1 was significantly associated with the birth weight of Hu sheep females. In addition, G > A mutation at s64890.1 was significantly associated with the yearling weight of Hu sheep females. The population verification results of the G3 generation were consistent with the GWAS results of G1 and G2, which proved the credibility of the GWAS results and showed that the mutation site was significantly related to the BW in the Hu sheep population. Combined with the results of the previous correlation analysis with OARX_76354330.1 and s64890.1 on the BW traits, these mutations could be identified as functional SNPs for the growth traits of Hu sheep as a molecular marker for later breeding.

During the growth and development, skeletal muscle is one of the important factors affecting the BW of Hu sheep. Therefore, we tested the differences in expression of the candidate genes in the longissimus dorsi and biceps femoris muscles, which rapidly develop during the growth period of the Hu sheep. There were significant differences in the expression of *CAPN6* in the biceps femoris and longissimus dorsi muscles of weaning (60-day) and 6-month sheep, which proved that during the growth process of Hu sheep, *CAPN6* was involved in the development of skeletal muscles. Recent studies have also identified SNP sites in *CAPN* that significantly affect sheep BW (Reena, 2014; Mahrous et al., 2016). Hence, *CAPN6* can be used as a candidate gene for BW traits in future research.

Taken together, the results of this study identified candidate genes related to the BW traits of Hu sheep, which will greatly enhance the breeding process by using the related SNPs for the early selection of Hu sheep.

In summary, four SNPs identified by GWAS were associated with the BW of Hu sheep, and *CAPN6*, *ITGA11*, and *SCMH1* can be used as candidate genes for the BW of Hu sheep. During the subsequent verification of significant SNP loci in the Hu sheep G3 generation nucleus herd, an additional nine SNPs were detected in the significant SNP regions. Three SNPs significantly associated with Hu sheep BW were identified. OARX_76354330.1 and s64890.1 were identified as functional SNPs for the growth traits of Hu sheep. *CAPN6* as a candidate gene was significantly different in the biceps femoris and longissimus dorsi muscles of weaning (60-day) and 6-month sheep. This phenomenon would facilitate the discovery of causal variants for BW and contribute toward the marker-assisted selection breeding of Hu sheep.

## Data Availability Statement

Data supporting this study has been deposited in GEO – accession number GSE152717.

## Ethics Statement

The animal study was reviewed and approved by All procedures performed in studies involving animals were conducted according to the Guidelines for the Care and Use of Animals of Zhejiang Academy of Agricultural Sciences, 2017. The study was approved by the Ethical Committee of Zhejiang Academy of Agricultural Sciences, Hangzhou, China.

## Author Contributions

FS, XS, and YJ conceived the project. YC performed the study and wrote the manuscript. HS, JJ, PX, and JW participated in the analysis of data. YJ is the leader of the project. All authors read and approved the final manuscript.

## Conflict of Interest

The authors declare that the research was conducted in the absence of any commercial or financial relationships that could be construed as a potential conflict of interest.

## Footnotes

1 http://www.animalgenome.org/QTLdb

2 http://www.maizegenetics.net

3 http://genome.ucsc.edu/

## References

[B1] Al-Mamun H. A. Kwan P. Clark S. A. Ferdosi M. H. Tellam R. Gondro C. (2015). Genome-wide association study of body weight in Australian Merino sheep reveals an orthologous region on OAR6 to human and bovine genomic regions affecting height and weight. *Genet. Select. Evol. GSE* 47:66. 10.1186/s12711-015-0142-4 26272623 PMC4536601

[B2] Buzanskas M. E. Grossi D. A. Ventura R. V. Schenkel F. S. Sargolzaei M. Meirelles S. L. (2014). Genome-wide association for growth traits in Canchim beef cattle. *PLoS One* 9:e94802. 10.1371/journal.pone.0094802 24733441 PMC3986245

[B3] Dekkers J. C. (2004). Commercial application of marker- and gene-assisted selection in livestock: strategies and lessons. *J. Anim. Sci.* 82(E–Suppl.), E313–E328. 10.2527/2004.8213_supplE313x 15471812

[B4] Ebrahimi F. Gholizadeh M. Rahimi-Mianji G. Farhadi A. (2017). Detection of QTL for greasy fleece weight in sheep using a 50 K single nucleotide polymorphism chip. *Trop. Anim. Health Product.* 49 1657–1662. 10.1007/s11250-017-1373-x 28801813

[B5] Gebreselassie G. Berihulay H. Jiang L. Ma Y. (2019). Review on genomic regions and candidate genes associated with economically important production and reproduction traits in sheep (*Ovies aries*). *Animals* 10:33. 10.3390/ani10010033 31877963 PMC7022721

[B6] Ghasemi M. Zamani P. Vatankhah M. Abdoli R. (2019). Genome-wide association study of birth weight in sheep. *Animal* 13 1797–1803. 10.1017/s1751731118003610 30616710

[B7] Hong J. M. Teitelbaum S. L. Kim T. H. Ross F. P. Kim S. Y. Kim H. J. (2011). Calpain-6, a target molecule of glucocorticoids, regulates osteoclastic bone resorption via cytoskeletal organization and microtubule acetylation. *J. Bone Miner. Res.* 26 657–665. 10.1002/jbmr.241 20814968 PMC3179291

[B8] Hu Z. L. Park C. A. Wu X. L. Reecy J. M. (2013). Animal QTLdb: an improved database tool for livestock animal QTL/association data dissemination in the post-genome era. *Nucleic Acids Res.* 41 D871–D879. 10.1093/nar/gks1150 23180796 PMC3531174

[B9] Lander E. Kruglyak L. (1995). Genetic dissection of complex traits: guidelines for interpreting and reporting linkage results. *Nat. Genet.* 11 241–247. 10.1038/ng1195-241 7581446

[B10] Luo L. Yang X. Takihara Y. Knoetgen H. Kessel M. (2004). The cell-cycle regulator geminin inhibits Hox function through direct and polycomb-mediated interactions. *Nature* 427 749–753. 10.1038/nature02305 14973489

[B11] Ma X. Guan L. Xuan J. Wang H. Yuan Z. Wu M. (2016). Effect of polymorphisms in the CAMKMT gene on growth traits in Ujumqin sheep. *Anim. Genet.* 47 618–622. 10.1111/age.12455 27435482

[B12] Mahrous K. F. Hassanane M. S. Shafey H. I. Abdel Mordy M. Rushdi H. E. (2016). Association between single nucleotide polymorphism in ovine Calpain gene and growth performance in three Egyptian sheep breeds. *J. Genet. Eng. Biotechnol.* 14 233–240. 10.1016/j.jgeb.2016.09.003 30647620 PMC6299862

[B13] Margawati E. T. Raadsma H. W. Martojo H. Subandriyo M. (2006). Quantitative trait loci (QTL) analysis for production traits of birth weight and weight 360 days in backcross sheep. *Hayati J. Biosci.* 13 31–35. 10.1016/s1978-3019(16)30376-x

[B14] Matika O. Riggio V. Anselme-Moizan M. Law A. S. Pong-Wong R. Archibald A. L. (2016). Genome-wide association reveals QTL for growth, bone and in vivo carcass traits as assessed by computed tomography in Scottish Blackface lambs. *GSE* 48:11. 10.1186/s12711-016-0191-3 26856324 PMC4745175

[B15] Parajuli H. Teh M. T. Abrahamsen S. Christoffersen I. Neppelberg E. Lybak S. (2017). Integrin α11 is overexpressed by tumour stroma of head and neck squamous cell carcinoma and correlates positively with alpha smooth muscle actin expression. *J. Oral Pathol. Med.* 46 267–275. 10.1111/jop.12493 27699902 PMC5396328

[B16] Pei Y. F. Liu L. Liu T. L. Yang X. L. Zhang H. Wei X. T. (2019). Joint association analysis identified 18 new loci for bone mineral density. *J. Bone Miner. Res.* 34 1086–1094. 10.1002/jbmr.3681 30690781

[B17] Ptáček M. Ducháček J. Stádník L. Hakl J. Fantová M. (2017). Analysis of multivariate relations among birth weight, survivability traits, growth performance, and some important factors in Suffolk lambs. *Archiv fur Tierzucht* 60 43–50. 10.5194/aab-60-43-2017

[B18] Reena A. (2014). Identification of novel single nucleotide polymorphisms in candidate genes for mutton quality in Indian sheep. *Ann. Pharm. Franc.* 25 553–559. 10.5376/amb.2014.04.0001

[B19] Russell D. W. Sambrook J. (2001). *Molecular Cloning: A Laboratory Manual.* Cold Spring Harbor Laboratory, NY: Cold Spring Harbor Laboratory Press.

[B20] Smeland H. Y. Lu N. Karlsen T. V. Salvesen G. Reed R. K. Stuhr L. (2019). Stromal integrin α11-deficiency reduces interstitial fluid pressure and perturbs collagen structure in triple-negative breast xenograft tumors. *BMC Cancer* 19:234. 10.1186/s12885-019-5449-z 30876468 PMC6419843

[B21] Su X. Xiao D. Huang L. Li S. Ying J. Tong Y. (2019). MicroRNA alteration in developing rat oligodendrocyte precursor cells induced by hypoxia-ischemia. *J. Neuropathol. Exp. Neurol.* 78 900–909. 10.1093/jnen/nlz071 31403686

[B22] Takada Y. Isono K. Shinga J. Turner J. M. Kitamura H. Ohara O. (2007). Mammalian Polycomb Scmh1 mediates exclusion of Polycomb complexes from the XY body in the pachytene spermatocytes. *Development* 134 579–590. 10.1242/dev.02747 17215307

[B23] Tao L. He X. Y. Pan L. X. Wang J. W. Gan S. Q. Chu M. X. (2020). Genome-wide association study of body weight and conformation traits in neonatal sheep. *Anim. Genet.* 51 336–340. 10.1111/age.12904 31960458

[B24] Tomotsune D. Takihara Y. Berger J. Duhl D. Joo S. Kyba M. (1999). A novel member of murine Polycomb-group proteins, Sex comb on midleg homolog protein, is highly conserved, and interacts with RAE28/mph1 *in vitro*. *Diff. Res. Biol. Divers.* 65 229–239. 10.1046/j.1432-0436.1999.6540229.x 10653359

[B25] Tonami K. Kurihara Y. Aburatani H. Uchijima Y. Asano T. Kurihara H. (2007). Calpain 6 is involved in microtubule stabilization and cytoskeletal organization. *Mol. Cell. Biol.* 27 2548–2561. 10.1128/mcb.00992-06 17210638 PMC1899902

[B26] Tonami K. Kurihara Y. Arima S. Nishiyama K. Uchijima Y. Asano T. (2011). Calpain-6, a microtubule-stabilizing protein, regulates Rac1 activity and cell motility through interaction with GEF-H1. *J. Cell Sci.* 124(Pt 8), 1214–1223. 10.1242/jcs.072561 21406564

[B27] Wu P. Wang Y. Wu Y. Jia Z. Song Y. Liang N. (2019). Expression and prognostic analyses of ITGA11, ITGB4 and ITGB8 in human non-small cell lung cancer. *PeerJ* 7:e8299. 10.7717/peerj.8299 31875161 PMC6927340

[B28] Yasunaga S. Ohtsubo M. Ohno Y. Saeki K. Kurogi T. Tanaka-Okamoto M. (2013). Scmh1 has E3 ubiquitin ligase activity for geminin and histone H2A and regulates geminin stability directly or indirectly via transcriptional repression of Hoxa9 and Hoxb4. *Mol. Cell. Biol.* 33 644–660. 10.1128/mcb.00974-12 23207902 PMC3571339

[B29] Zhang L. Liu J. Zhao F. Ren H. Xu L. Lu J. (2013). Genome-wide association studies for growth and meat production traits in sheep. *PLoS One* 8:e66569. 10.1371/journal.pone.0066569 23825544 PMC3692449

[B30] Zhu L. Sun Y. Wu Q. Zhang C. Ling J. (2020). CAPN6 regulates uterine leiomyoma cell proliferation and apoptosis through the Rac1-dependent signaling pathway. *Ann. Clin. Lab. Sci.* 50 24–30. 32161009

