# Supplementary material for: Genome-Wide Association Study of Body Weights in Hu Sheep and Population Verification of Related Single-Nucleotide Polymorphisms
Source: Front Genet. 2020 Jul 3;11:588. doi: 10.3389/fgene.2020.00588 (PMC7350885; doi:10.3389/fgene.2020.00588)
Supplement: Supplementary file 1 [file Data_Sheet_1.PDF]

Supplementary Materials

1. Supplementary Figures and Tables

Table S1. Primers for PCR and direct sequencing

SNPs	Nearest gene Distance# (bp)	Chr	Primer sequence(5'→3')	Annealing degree(℃)	Amplification product length (bp)
s37755.1	*SCMH1* within	1	GGACACAATAGAGATGAGCAG<br>CTTCCTGCCCAAGAATTAC	53	490
s39389.1	*ITGA11* within	7	CACTGTGGCCTGTGTTTCA<br>CAGGGCCTTGACTTCCTGT	57	317
OARX_76354330.1	*CAPN6* within	27	CAACCACCAGCTGACCCAAGGGA<br>CAACTAATACGTGTGACAAAGCAA	55	347
s64890.1	*CAMTA1* -364270	12	CCAGGTCCCTATTATCTGTG<br>TCTGCTGCTAATCACTTCCT	57	404

Table S2. Primers for qPCRto detection of candidate genes

Candidate genes	Chr	Primer sequence(5'→3')	Annealing degree(℃)	Amplification product length (bp)
SCMH1	1	GAAGCTCTGCCACAACCTTC<br>GTGCTGACAAGATTGGTGGG	60	213
ITGA11	7	GAGCCATCCAAGATCAACATC<br>CTTCACGTAGTCAGCGGTATCC	60	291
CAPN6	27	TGGCTGTTCAGGAGTGTCACT<br>CTCATTCATGGAGGTGGAGA	60	203

Table S3.SNPs associated with body weight traits and mutation types and methods in Hu sheep

Nearest gene Distance#(bp)	SNP loci name	Mutagenesis	Mutation type
SCMH1 within	mutation at 68 bp upstream of s37755.1	T>C	transition
	mutation at 7 bp upstream of s37755.1	C>T	transition
	s37755.1	G>A	transition
	mutation at 29bp downstream of s37755.1	A>G	transition
ITGA11 within	mutation at 63 bp upstream of s39389.1	T>C	transition
	s39389.1	T>C	transition
	mutation at 37 bp downstream of s39389.1	G>A	transition
CAPN6 within	OARX_76354330.1	T>A	transition
	mutation at 27 bp downstream of OARX_76354330.1	A>G	transition
CAMTA1 -364270	mutation at 68 bp upstream of s64890.1	T>C	transition
	s64890.1	G>A	transition

	mutation at 34 bp downstream of s64890.1	T>C	transition
	mutation at 59 bp downstream of s64890.1	G>A	transition

Table S4. Genetic parameters of SNPs associated with body weight traits in Hu sheep

Nearest gene<br>Distance# (bp)	SNP loci name	Ne	Shannon information content	Site heterozygosity
SCMH1<br>within	mutation at 68 bp upstream of s37755.1	2.0000	0.6931	0.5000
	mutation at 7 bp upstream of s37755.1	1.2515	0.3534	0.2009
	s37755.1	1.1813	0.2878	0.1535
	mutation at 29bp downstream of s37755.1	2.0000	0.6931	0.5000
ITGA11<br>within	mutation at 63 bp upstream of s39389.1	1.4183	0.4711	0.2949
	s39389.1	1.1197	0.2176	0.1069
	mutation at 37 bp downstream of s39389.1	1.1197	0.2176	0.1069
CAPN6<br>within	OARX_76354330.1	1.5141<br>1.5077	0.5228<br>0.5196	0.3395<br>0.3367
	mutation at 27 bp downstream of OARX_76354330.1	1.5141<br>1.5077	0.5228<br>0.5196	0.3395<br>0.3367
CAMTA1<br>-364270	mutation at 68 bp upstream of s64890.1	1.8538	0.6532	0.4606
	s64890.1	1.9905	0.6908	0.4976

	mutation at 34 bp downstream of s64890.1	1.8538	0.6532	0.4606
	mutation at 59 bp downstream of s64890.1	1.1418	0.2445	0.1242

Table S5. Population genetic analysis of SNPs related to body weight traits in Hu sheep

Gene	SNP loci name	Genotype	Numbers	Genotype frequencies	Allele	Allele frequencies	PIC
SCMH1 within	mutation at 68 bp upstream of s37755.1	TT	48	0.2364	T	0.4975	0.3749
		TC	106	0.5221			
		CC	49	0.2413	C	0.5025	
	mutation at 7 bp upstream of s37755.1	CC	159	0.7832	C	0.8867	0.1807
		CT	42	0.2068			
		TT	2	0.0098	T	0.1133	
	s37755.1	GG	170	0.8374	G	0.9163	0.1416
		GA	30	0.1447			
		AA	2	0.0098	A	0.0837	
	mutation at 29bp downstream of s37755.1	AA	49	0.2413	A	0.5000	0.3750
		AG	105	0.5172			
		GG	49	0.2413	G	0.5000	
ITGA11 within	mutation at 63 bp upstream of s39389.1	TT	134	0.6600	T	0.8202	0.2514
		TC	65	0.3400			

		CC	4	0	C	0.1798	
	s39389.1	TT	180	0.8866	T	0.9433	0.1012
		TC	23	0.1133			
		CC	0	0	C	0.0567	
	mutation at 37bp downstream of s39389.1	GG	180	0.8866	G	0.9433	0.1012
		GA	23	0.1133			
		AA	0	0	A	0.0567	
CAPN6 within	mutation at 27 bp upstream of OARX_76354330.1	TT	121	0.596059	T	0.7833	0.2818
		TA	76	0.374384			
		AA	6	0.029557	A	0.2167	
	OARX_76354330.1	AA	121	0.596059	A	0.7857	0.2800
		AG	77	0.37931			
		GG	5	0.024631	G	0.2143	
CAMTA1 -364270	mutation at 68 bp upstream of s64890.1	TT	85	0.4187	T	0.6404	0.3545
		TC	90	0.4433			
		CC	28	0.1379	C	0.3596	
	s64890.1	GG	43	0.2118	G	0.4655	0.3738

		GA	103	0.5073			
		AA	57	0.2807	A	0.5345	
	mutation at 34 bp downstream of s64890.1	TT	85	0.4187	T	0.6404	0.3545
		TC	90	0.4433			
		CC	28	0.1379	C	0.3596	
	mutation at 59 bp downstream of s64890.1	GG	176	0.8669	G	0.9335	0.1164
		GA	27	0.1330			
		AA	0	0	A	0.0665	
